# Part Affinity Fields and CoordConv for Detecting Landmarks of Lumbar Vertebrae and Sacrum in X-ray Images

**DOI:** 10.3390/s22228628

**Published:** 2022-11-09

**Authors:** Chang-Hyeon An, Jeong-Sik Lee, Jun-Su Jang, Hyun-Chul Choi

**Affiliations:** 1 Intelligent Computer Vision Software Laboratory (ICVSLab), Department of Electronic Engineering, Yeungnam University, 280 Daehak-Ro, Gyeongsan 38541, Gyeongbuk, Korea; 2 Korea Institute of Oriental Medicine, 1672 Yuseong-daero, Yuseong-gu, Daejeon 34054, South Chungcheong, Korea

**Keywords:** X-ray image, spine detection, landmark detection, random spine cutout, CoordConv, part affinity fields

## Abstract

With the prevalence of degenerative diseases due to the increase in the aging population, we have encountered many spine-related disorders. Since the spine is a crucial part of the body, fast and accurate diagnosis is critically important. Generally, clinicians use X-ray images to diagnose the spine, but X-ray images are commonly occluded by the shadows of some bones, making it hard to identify the whole spine. Therefore, recently, various deep-learning-based spinal X-ray image analysis approaches have been proposed to help diagnose the spine. However, these approaches did not consider the characteristics of frequent occlusion in the X-ray image and the properties of the vertebra shape. Therefore, based on the X-ray image properties and vertebra shape, we present a novel landmark detection network specialized in lumbar X-ray images. The proposed network consists of two stages: The first step detects the centers of the lumbar vertebrae and the upper end plate of the first sacral vertebra (S1), and the second step detects the four corner points of each lumbar vertebra and two corner points of S1 from the image obtained in the first step. We used random spine cutout augmentation in the first step to robustify the network against the commonly obscured X-ray images. Furthermore, in the second step, we used CoordConv to make the network recognize the location distribution of landmarks and part affinity fields to understand the morphological features of the vertebrae, resulting in more accurate landmark detection. The proposed network was evaluated using 304 X-ray images, and it achieved 98.02% accuracy in center detection and 8.34% relative distance error in corner detection. This indicates that our network can detect spinal landmarks reliably enough to support radiologists in analyzing the lumbar X-ray images.

## 1. Introduction

Recently, due to the global population’s aging, there has been a gradual increase in the proportion of the elderly. As a result, many degenerative diseases, such as osteoporosis, arthritis, and muscular regression, are becoming more common. Furthermore, many spine-related disorders are occurring because of these degenerative diseases. The spine is important in our bodies because it protects the central and peripheral nerves. Furthermore, once a spinal problem starts, it is hard to recover completely. Thus, it is critical to diagnose it early and to receive appropriate therapy. In this diagnosis, clinicians can use Computed Tomography (CT), Magnetic Resonance Imaging (MRI), and X-ray images. However, obtaining CT or MRI images consumes much time and money. Therefore, for initial diagnosis, many clinicians use X-ray images, which are relatively inexpensive and easy to acquire. In the case of X-ray images, however, tissues or shadows of other bones overlap, making it difficult to diagnose. As a result, numerous deep-learning-based vertebral analysis methods have been proposed that assist in diagnoses, such as automatic vertebral landmarks’ detection or vertebral segmentation. In particular, the detection of vertebral landmarks is important for the quantitative analysis of spine alignments, along with the diagnosis of spondylolisthesis, scoliosis, compression factor, and degenerative change.

Among the previous methods, some works [1,2,3,4] adopted a one-stage process to perform detection or segmentation in spine X-ray images. However, because X-ray images are frequently occluded, identifying all landmarks in a single process is exceptionally challenging. Therefore, most of the recently proposed methods [5,6,7] adopted a two-stage process that determines vertebral regions to crop and then performs spinal detection or segmentation. Kim et al. [5] used the predicted center of each lumbar vertebra to determine the vertebral region and performed segmentation. However, Kim et al. did not consider the characteristic of the X-ray sufficiently, which is generally occluded; occasionally, the center of the first lumbar vertebra was not predicted adequately. Additionally, since Kim et al. did not segment the sacrum, spondylolisthesis diagnosis at the fifth lumbar vertebra (L5)—S1 level is impossible. Contrary to Kim et al., Cina et al. [6] performed landmark detection in both S1 and lumbar vertebrae and, when determining the vertebral region, using an expanded bounding box generated by roughly predicted landmarks of each vertebra. However, Cina et al. had difficulty accurately detecting every landmark as the already mentioned problem of one-stage detection, and once a single landmark was improperly detected, adequately making the bounding box was impossible.

**Figure 1:**
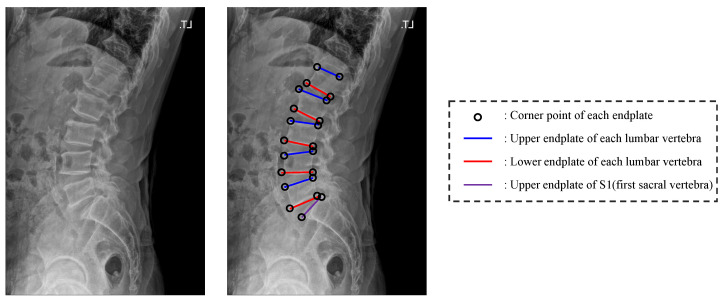
Illustration for the corner point and upper and lower end plate of each vertebra.

In this paper, we propose a network that detects four corner points of the upper end plate and lower end plate of each lumbar vertebra and two corner points of the upper end plate of S1 in lateral lumbar X-ray images (Figure 1). Here, we excluded the lower end plate of S1 since it is often difficult to localize in the X-ray image. Our proposed network detects the center of each vertebra based on the confidence maps, and we performed landmark detection in cropped vertebral images using the detected centers. For detecting centers, we adopted Pose-Net [5] and increased the kernel size of some convolution filters to enable accurate center detection with broad receptive fields. In addition, a novel augmentation technique called random spine cutout was applied, which randomly cuts the vertebra employing a conventional cutout [8] to precisely localize the center, even when the X-ray image is obscured. In the landmark detection process, we utilized M-Net [9] and used CoordConv [10] instead of conventional convolution in the encoding layers, which helps M-Net understand the distribution of positions between each landmark, enabling more accurate detection. Further, we let M-Net recognize the appearance characteristics of the vertebra by making M-Net predict part affinity fields (PAFs) when detecting landmarks. At this time, we utilized two M-Nets, which share a similar structure, for detecting landmarks of the lumbar vertebrae and S1 because of the different shapes between the S1 and lumbar vertebrae. As a result, the proposed network achieved 98.02% accuracy in center detection and an 8.34% relative error rate in landmark detection.

Our contributions are summarized as follows:

- We propose a novel network to detect landmarks of the lumbar vertebrae and S1 specialized in X-ray images.
- The proposed network has a wide range of receptive fields, and it is capable of precise vertebral center localization.
- In X-ray images, we demonstrate that random spine cutout is more efficient than the conventional cutout.
- We show that using CoordConv helps the network to localize an invisible landmark by learning the location distribution of landmarks.
- Furthermore, we demonstrate that learning the morphological properties of the vertebra by additionally predicting PAFs is effective in improving spinal landmark detection’s accuracy with a slightly greater computational cost.

## 2. Related Work

### 2.1. Pose Estimation

Pose estimation is a task for localizing the joints of the body. DeepPose [11] was the first approach that utilized deep learning in pose estimation. DeepPose used a regressor that predicts the target joint by a sequential process. However, DeepPose did not consider the spatial connection between joints and performed pose estimation sequentially, which has the disadvantage of poor prediction accuracy and slow learning and inference. Thomson et al. [12] adopted a heat map (i.e., confidence map of joint) into pose estimation to solve the problem of inaccuracy in a high-precision region and made the estimation faster. Furthermore, it prevents improper estimations using the correlation between joints. However, it has the limitation of predicting only one person’s pose in the image. Cao et al. [13] allowed multiple pose estimations within an image in a bottom-up manner to predict all joints in the image and then connected all joints of each person. To properly link all joints of each person based on the relationship between joints, Cao et al. predicted part affinity fields when joining joints for each individual. Moreover, Cao et al. performed real-time pose estimation by fast inference. HRNet [14] employed a top-down approach, first recognizing a person and then detecting all joints from that person. The top-down method is slower than the bottom-up method because the top-down method recognizes humans first before estimating the pose, but the top-down method can estimate poses with higher accuracy. In addition, HRNet maintained the resolution of the input image, used multi-resolution in parallel, and enabled detailed pose estimation with a high-resolution image. Because of these advantages, it showed good performance not only in pose estimation, but also in detection and segmentation [14].

### 2.2. Spinal Segmentation and Detection

In the lumbar X-ray images, various deep-learning-based segmentation approaches [3,4,5] for spinal diagnosis have been proposed. Cho et al. [3] presented a method for automatically calculating the lumbar lordosis angle by segmenting the sacrum and lumbar vertebrae using U-Net [15] and the DSC loss [16]. However, it was unsuccessful for occlusions in X-ray images since the segmentation process was carried out simply in the single stage. MBNet [4] recovered the lost information that occurred in the downsampling process during the upsampling process by utilizing a feature fusion module [17] with U-Net. Additionally, it improved the segmentation performance by predicting the parameters required for lumbar vertebra inspection. However, there is a problem that network learning is possible only when there is a ground truth for the parameters. Kim et al. [5] proposed a hierarchical segmentation network that detects the centers of lumbar vertebrae based on the confidence map frequently used in pose estimation [12,13,14], splits the vertebral region, and then performs segmentation and fine-tuning.

Along with many proposed segmentation methods, many landmark detection methods [1,2,6,7,18] have also been proposed. Yi et al. [1] detected the spinal centers and then localized the landmarks by predicting an offset between the center and the landmarks. Yeh et al. [2] received the whole-spine local view radiographs and ensembled two models to detect all spinal landmarks. However, both Yi et al. and Yeh et al. performed detection in the downscaled full spinal image rather than in each vertebral image, making landmark localization difficult. Cina et al. [6] proposed a network that detects the landmarks of the lumbar vertebrae and nearby vertebrae (T9-12, S1) after cropping each vertebra based on the previously obtained rough landmarks. However, proper cropping of each vertebra is hard when roughly detected landmarks are not clearly localized. Zhang et al. [18] detected spinal landmarks and used part affinity fields to calculate a more accurate Cobb angle. Khanal et al. [7] split each vertebra image using Faster-RCNN [19] and then performed landmark detection. At this time, however, bottlenecks occurred during the region proposal process of Faster-RCNN, resulting in a delay in landmark detection.

Numerous methods using MRI [20,21] and CT [22] images have also been proposed in addition to X-ray images. DeepSPINE [21] detects the centers of the lumbar vertebrae and segments the lumbar vertebrae so it could support the diagnosis of lumbar spinal stenosis. Furthermore, SpineOne [20] determines the center of the lumbar vertebrae and the discs in MRI images and could assist in diagnosing degenerative discs and vertebrae. Payer et al. [22] presented a method that performs vertebral center detection and segmentation in CT images. However, MRI and CT images have the problem of requiring much time and money to shoot. In addition, since these observe the spine relatively more easily than X-ray images, developing X-ray image analysis assisting methods is more needed.

## 3. Method

In this section, we first explain the two main components of our network. Furthermore, we describe the learning strategy for our network. In summary, our network includes the significant role of the following: (1) Pose-Net: predicts the centers of the lumbar vertebrae and the upper end plate of S1 (first sacral vertebra). (2) M-Net: detects the landmarks of each vertebra. Throughout this paper, we refer to the upper end plate of S1 as the sacrum, and from top to bottom, we call the lumbar vertebrae as L1 to L5.

### 3.1. Network Structure

It is highly challenging to accurately identify landmarks from X-ray images using a straightforward convolutional inference procedure since the X-ray image is generated in a 2-dimensional image, overlapping the tissues or shadows of numerous bones. Therefore, first, we chose the 2-stage method, which finds the centers of the lumbar vertebrae and sacrum first, then crops each vertebra based on the center and, finally, detects the landmarks for each cropped image.

**Figure 2:**
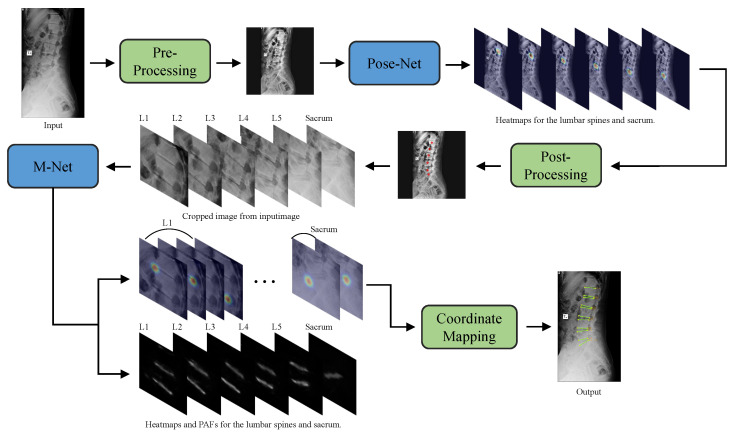
An overview of the proposed network. The blue boxes denote the neural network and the green boxes denote the algorithmic process.

Figure 2 represents the overall flow of our proposed network. On a high level, it consists of pre-processing the input image before applying Pose-Net, after which we obtained the center coordinates of each vertebra using Pose-Net and post-processing. Then, we cropped each vertebra image from a zero-padded input image to feed as the input for M-Net to achieve landmark detection. These locally detected landmarks are then mapped to the input image using coordinate mapping.

### 3.2. Detecting Centers of the Lumbar Vertebrae and the Sacrum

**Pre-Processing:** To save the computing costs, we resized the input images to 512 × 512 pixels. At this point, we applied zero padding to handle multiple resolutions of input images, ensuring that the aspect ratio remains the same. Additionally, all images were subjected to Gaussian blurring and contrast limited adaptive histogram equalization (CLAHE) [23] to minimize noise and ensure the distinction between the spine from the background.

**Figure 3:**
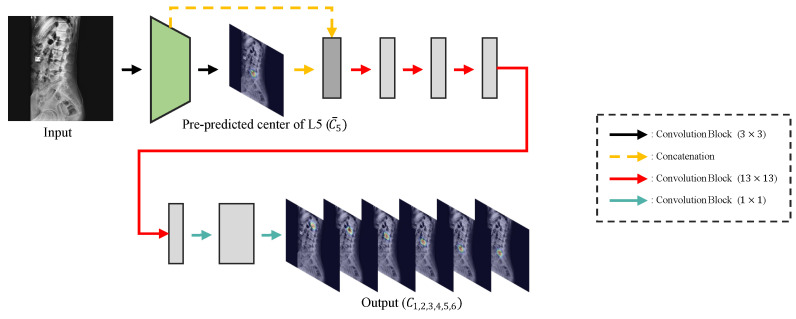
Structure of our Pose-Net.

**Detecting the center:** In pose estimation, estimating the probability of the target joint location in the form of the confidence map [12,13,14] showed an outstanding performance compared to utilizing the regressor [11]. Therefore, following Kim et al. [5], we adopted Pose-Net to detect the centers of the vertebrae based on the confidence map. We modified Pose-Net by expanding the receptive fields of the convolutional layers by increasing the 7 × 7 size of the convolution filter to 13 × 13. Consequently, this decreased the observed outliers since the increased visibility of nearby vertebrae helps analyze better while predicting the centers of vertebrae. Furthermore, we also modified Pose-Net to detect the landmarks of the sacrum. Kim et al. produced center coordinates of the lumbar vertebrae after post-processing the generated confidence map of 1 channel. However, in our case, we not only detected the centers of lumbar vertebrae, but the center of the sacrum as well, and due to the proximity of S1 and L5, it is challenging to extract each center coordinate from the confidence map of 1 channel. Thus, by dividing the confidence maps $C\in R^{6\times 64\times 64}$ for each center of the lumbar vertebrae and the sacrum from 1 channel to 6 channels ($C_{i=1,2,3,4,5,6}$), we made it simpler to extract each center coordinate. $C_{i=1,\ldots ,6}$ represents the confidence maps of each center for L1–L5 and the sacrum in order. Additionally, we also changed the vanilla convolutional blocks (convolution, normalization, and activation) of the previous Pose-Net to the pre-activation design (normalization, activation, and convolution), where for the normalization layer, we used instance normalization [24] along with ReLU in the activation layer. The detailed structure of Pose-Net is shown in Figure 3. All the abstracted parts are the same as the structure of the previous Pose-Net.

**Training procedures:** To robustly train our Pose-Net with a limited dataset, we suggest a random spine cutout augmentation technique, which randomly cuts the vertebra, to consider the frequent occlusion in X-ray images. Furthermore, we used two existing augmentation techniques. First, to deal with the changes in visual quality, such as brightness and contrast, we employed random brightness and contrast adjustment augmentations. Second, since each vertebra has a different appearance, we randomly applied rotation, scaling, and translation augmentations.

Cutout [8] is primarily used in classification tasks to enhance a classifier’s performance by masking a portion of an image while training the classifier. However, this conventional cutout cuts the arbitrary region in the image, and it is unsuitable for use in the lumbar X-ray image, which has a large background area, rather than the spine. Therefore, we propose a new augmentation method named random spine cutout (RSC), which randomly cuts the lumbar vertebrae utilizing the conventional cutout.

**Figure 4:**
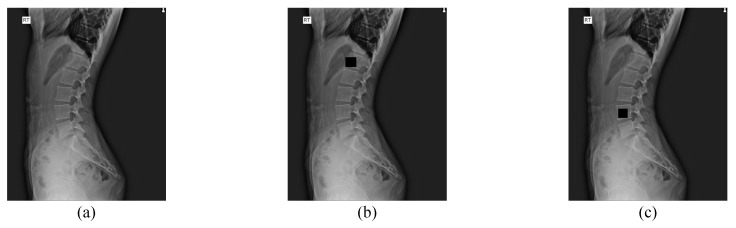
Example of random spine cutout. (**b**,**c**) are results of the random spine cutout performed on (**a**).

In RSC, as shown in Figure 4, a vertebra is randomly selected from the lumbar vertebrae (L1–L4) and masks to a zero value. We performed RSC only among the often occluded vertebrae (L1, L2, L3, L4), whose average pixel values inside the vertebra are low, as shown in Table 1. Additionally, if the whole vertebra region is cut, it might be infeasible to find the center of that vertebra. Thus, we randomly cut the region only 60% of the vertebra’s width and length, which was an experimentally determined value.

Our Pose-Net pre-predicts a confidence map ${\bar{C}}_{5}$ for the center of L5 in the internal layer of Pose-Net, similar to Kim et al. [5], and concatenates it with the feature of Pose-Net’s internal layer to output confidence maps $C_{i=1,2,3,4,5,6}$ for each center of the lumbar vertebrae and sacrum.

To train Pose-Net, we employed the following ground truth of center confidence maps ${\hat{C}}_{i=1,2,3,4,5,6}$ based on the ground truth of center coordinates ${\hat{c}}_{i=1,2,3,4,5,6}$ of the lumbar vertebrae and sacrum.

(1) $${\hat{C}}_{i}\left(x\right)=exp\left(-\frac{||x-{\hat{c}}_{i}{\left|\right|}_{2}^{2}}{2\sigma^{2}}\right)$$

where *x* represents a pixel position in $\hat{C}$ and σ is given as 1/2 of the L5 height, which is the value when Pose-Net performs best. The loss function $L_{Pose}$ using ${\hat{C}}_{1,\ldots ,6}$, ${\bar{C}}_{5}$, and $C_{i=1,\ldots ,6}$ was used to train our Pose-Net.

(2) $$L_{pre}=\left|\right|{\hat{C}}_{5}-{\bar{C}}_{5}{\left|\right|}_{2}$$

(3) $$L_{final}=\frac{1}{6}\sum_{i=1}^{6}\left|\right|{\hat{C}}_{i}-C_{i}{\left|\right|}_{2}$$

(4) $$L_{Pose}=L_{pre}+L_{final}$$

**Figure 5:**
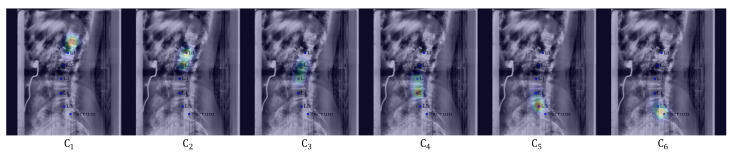
Failure example. Blue circles and labels denote the ground truth of each vertebra. From left to right, the confidence maps for the centers of L1, L2, L3, L4, L5, and the sacrum are displayed with the input images, respectively.

**Figure 6:**
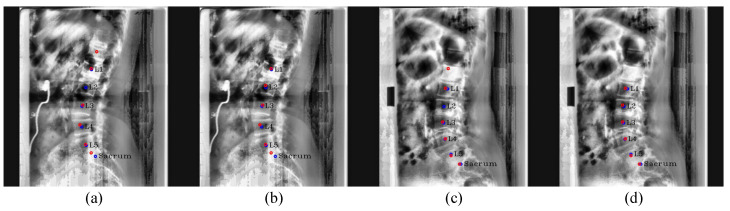
Post-processing results. (**a**,**c**) are the resulting images before performing post-processing, and (**b**,**d**) are the resulting images after performing post-processing on (**a**,**c**), respectively. The red circles denote the predicated centers of L1, L2, L3, L4, L5, and the sacrum in order from the top, and the blue circles and labels denote the ground truth of the center of each vertebra.

**Post-processing:** For Figure 5, when Pose-Net incorrectly predicts the center of the upper vertebra of L1 (T12) rather than L1 in a channel $C_{1}$, where L1 should have been predicted, the successive target centers from $C_{2}$ to $C_{4}$ are also not properly detected. At this time, if we only selected the maximum point of each confidence map ($max_{1},max_{2},max_{3},max_{4},max_{5},$$max_{6}$) as the center of each vertebra, there were cases where we would inaccurately extract the centers of some lumbar vertebrae, as shown in Figure 6a,c. To solve this problem, we used post-processing by taking advantage of the fact that all confidence maps for the centers of all lumbar vertebrae and the sacrum exist. First of all, we computed the distance $d_{i=1,\ldots ,5}$ between the maximum points of each confidence map to determine whether $C_{i=1,\ldots ,6}$ is the same case as Figure 6a,c. $d_{i=1,\ldots ,5}$ is calculated as $||max\left(C_{i}\right),max\left(C_{i+1}\right){\left|\right|}_{2}$ using the maximum points of $C_{i}$ and $C_{i+1}$. Then, we calculated an average value $mean(d_{except(max(d))})$ of the $d_{i=1,\ldots ,5}$, excluding the maximum value of $d_{i=1,\ldots ,5}$. If max(d) > $mean(d_{except(max(d))})$× 1.4, it was determined that part of the center coordinates of the vertebrae were not extracted exactly, and post-processing was carried out. Here, $mean(d_{except(max(d))})$× 1.4 was the maximum distance between two local maximum points of the center confidence map in our results.

**Figure 7:**
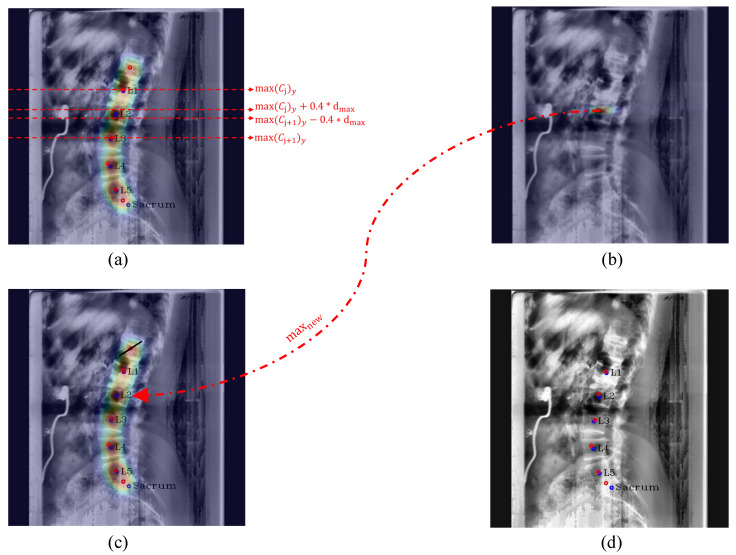
Post-processing procedure. (**a**) denotes the boundary lines to make the value of the confidence map zero, (**b**) denotes the generated confidence map of the unextracted center by using the previous boundary lines, (**c**) denotes the process of removing the incorrectly extracted center point of T12 and inserting the correct center point, and (**d**) denotes the final center points after post-processing. The red circles denote the predicated centers of L1, L2, L3, L4, L5, and the sacrum in order from the top, and the blue circles and labels denote the ground truth of the center of each vertebra.

The post-processing process is detailed in Algorithm 1 and Figure 7. In Algorithm 1, 0.4 in the equation $p_{y}$ > $max\left(C_{j+1}\right)-0.4\times d_{max}$ was an experimentally obtained value, which can properly derive the center confidence map of the vertebra whose center was not extracted. As a result, thanks to the post-processing, we can remove the T12 center point from the improperly extracted center points and properly extract the unextracted center from the channelwise summed confidence map.
**Algorithm 1** Post-processing.Obtain $C_{sum}$ by channelwise summation of all confidence maps of centersCalculate the y-coordinate distance $d_{max}$ between the furthest maximum points ($max(C_{j})$ and $max(C_{j+1})$)**for** The y-coordinate value of each pixel $p_{y}$ in $C_{sum}$ **do**      **if** $p_{y}$ < $max{\left(C_{j}\right)}_{y}+0.4\times d_{max}$ **then**            The value of *p*← 0      **end if**      **if** $p_{y}$ > $max{\left(C_{j+1}\right)}_{y}-0.4\times d_{max}$ **then**            The value of *p*← 0      **end if****end for**Obtain a new maximum point $max_{new}$ in $C_{sum}$Remove the top of the maximum point from {$max_{1},\ldots ,max_{6}$}Insert the $max_{new}$ between the maximum point of $C_{j}$ ($max_{j}$) and the maximum point of $C_{j+1}$ ($max_{j+1}$) in the maximum point setObtain the final center locations {$max_{2},\ldots ,max_{j},max_{new},max_{j+1},\ldots ,max_{6}$}

### 3.3. Detecting Landmarks of the Lumbar Vertebrae and the Sacrum

**Pre-processing:** Using the center coordinates $c_{i=1,2,3,4,5,6}$ of each vertebra determined by post-processing the Pose-Net results, we cropped each vertebral region from the zero-padded original image. The cropped images were centered on the center $c_{i}$ corresponding to each vertebra and were cropped into a square form by calculating the height *H* and width *W* using the y-axis distance between the center coordinates of the neighboring vertebrae as given in Equation (5).

(5) $$H=W=\left\{\begin{array}{cc} 3/2|{\left(c_{i}\right)}_{y}-{\left(c_{i+1}\right)}_{y}|, & i=1\\ 3/4(|{\left(c_{i-1}\right)}_{y}-{\left(c_{i}\right)}_{y}|+|{\left(c_{i}\right)}_{y}-{\left(c_{i+1}\right)}_{y}|), & i=2,3,4\\ 3/2|{\left(c_{i-1}\right)}_{y}-{\left(c_{i}\right)}_{y}|, & i=5\\ 3/2|{\left(c_{i-2}\right)}_{y}-{\left(c_{i-1}\right)}_{y}|, & i=6 \end{array}\right.$$

where ${\left(c_{i}\right)}_{y}$ denotes the y-coordinate of $c_{i}$. Due to the extremely wide variance of the distance between the centers of the sacrum and L5, the H and W of the sacrum area were determined using the y-axis distance between the L4 and L5 centers. After cropping, we resized the cropped image to 256 × 256 pixels and applied Gaussian blurring so that it could be used as an input for M-Net.

**Figure 8:**
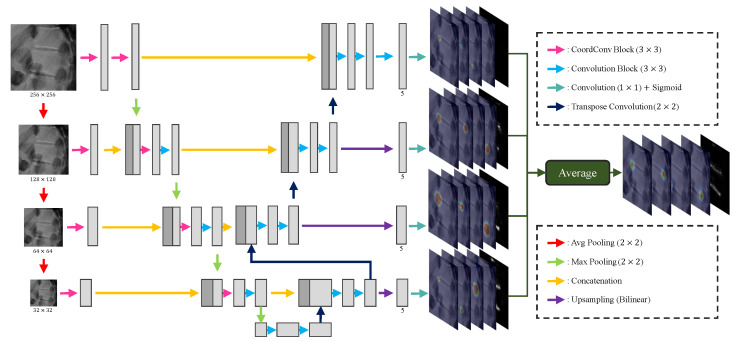
An overview of our proposed M-Net. In each resolution, the channel of all features except the channel (5) of the last feature is the same as Kim et al. [5].

**Detecting landmarks:** Based on U-Net [15], which is mostly utilized in the segmentation task of medical images, M-Net [9] can use a range of receptive fields utilizing multi-scale inputs and shows superior performance compared to U-Net [5,9]. High segmentation performance translates into significant spatial comprehension of a given object, which means M-Net can also show good performance in the task of detecting landmarks. Therefore, we utilized the M-Net structure for the landmark detection of vertebrae, and Figure 8 shows the detailed design. In Figure 8, we modified all convolution blocks to the same pre-activation design as our Pose-Net. During landmark detection, we used two M-Nets with identical structures, except for the last layer, assuming the appearances of the lumbar vertebrae and sacrum are very different. In the case of the sacrum, which has two landmarks, M-Net outputs a 3-channel result including confidence maps of landmarks and part affinity maps.

**Figure 9:**
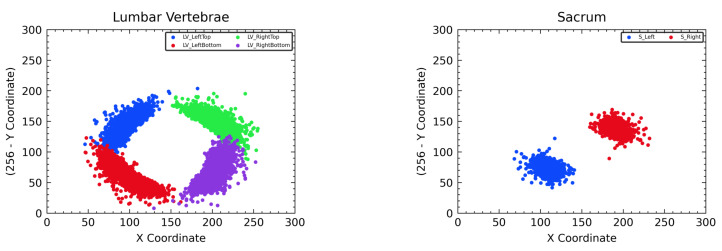
Distribution of all landmark coordinates in the cropped vertebra images for training M-Net. The left side represents the case of lumbar vertebrae, and the right side represents the case of the sacrum.

The CoordConv block in Figure 8 is the convolution block that uses CoordConv [10] instead of the conventional convolution. CoordConv performs the convolution operation after simply concatenating the coordinates corresponding to the position in the input feature normalized to the (−1,1) values with the input feature. This enables the translation-invariant convolution process to use pixel location information, leading to substantial performance gains in tasks such as object detection [10] and segmentation [25]. Therefore, we exploited the similarly positioned landmarks for each cropped vertebrae image, as shown in Figure 9, to allow M-Net with CoordConv to predict the exact location of the landmark based on the learned location distribution, even when the landmarks are occluded.

Part affinity fields (PAFs) is a concept introduced by the pose estimation task [13], which enables the network to connect all the joints of a particular person in an image by recognizing the connectivity of each joint found during pose estimation. We made M-Net learn the morphological information of vertebrae by predicting the PAFs. When training M-Net to learn PAFs, we enforced it to estimate the probability of a region with a linking segment $\bar{L_{t}R_{t}}$ of the top-left to top-right lumbar vertebral landmarks and a linking segment $\bar{L_{b}R_{b}}$ of the bottom-left to bottom-right lumbar vertebral landmarks. Similarly, in the case of the sacrum, the PAF is defined by a line segment that connects the two landmarks of the sacrum. Learning PAFs allows M-Net to estimate the shape of the vertebrae from the learned morphological information and recognize relations within PAFs (i.e., at the edges of PAFs are landmarks of lumbar vertebrae and the sacrum). In each lumbar vertebrae, $\bar{L_{t}R_{t}}$ and $\bar{L_{b}R_{b}}$ are similarly apart. $\bar{L_{t}R_{t}}$ is generally parallel to $\bar{L_{b}R_{b}}$), so that it can detect the proper landmarks when occlusion occurs in the X-ray image. Furthermore, PAFs can be employed in place of landmarks through post-processing.

**Training procedures:** For effective M-Net learning, we generated an input image of M-Net by cropping using the ground truth of the center coordinates from the zero-padded original input image, while, during inference, we used the center coordinates calculated through Pose-Net. In addition, we employed random scaling, rotation, brightness, and contrast adjustment augmentations, similar to training Pose-Net. Additionally, random translation augmentation was used to robustly respond to the difference between the center coordinates used in inference and training.

To train M-Net ($M^{L}$), which detects the landmarks of the lumbar vertebrae, we constructed the ground truth of confidence maps ${\hat{C}}_{i=1,2,3,4}^{L}$ of each landmark as given in Equation (6) using four ground truth landmarks ${\hat{l}}_{i=1,2,3,4}^{L}$ of each vertebra. Similarly, to train M-Net ($M^{S}$), which detects the landmarks of the sacrum, we constructed the ground truth of confidence maps ${\hat{C}}_{i=1,2}^{S}$ as given in the following Equation (7) using two ground truth of landmarks ${\hat{l}}_{i=1,2}^{S}$ of the sacrum.

(6) $${\hat{C}}_{i}^{L}\left(x\right)=exp\left(-\frac{||x-{\hat{l}}_{i}^{L}{\left|\right|}_{2}^{2}}{2\sigma^{2}}\right)$$

(7) $${\hat{C}}_{i}^{S}\left(x\right)=exp\left(-\frac{||x-{\hat{l}}_{i}^{S}{\left|\right|}_{2}^{2}}{2\sigma^{2}}\right)$$

where *x* represents a pixel position in $\hat{C}$. In Equation (6), σ is given as 1/10 of the average length of two lines diagonally connected to the landmarks of each vertebra, and in Equation (7), σ is given as 1/6 of the distance between two landmarks in each sacrum, defined experimentally. Additionally, Equations (8) and (9) show the ground truth of the PAFs we used for training $M^{L}$${\hat{P}}^{L}\in R^{W\times H}$ and training $M_{S}$${\hat{P}}^{S}\in R^{W\times H}$, respectively.

(8) $${\hat{P}}^{L}\left(x\right)=\left\{\begin{array}{cc} 1, & \mathrm{if}\;(x)\;\mathrm{on}\;\bar{L_{t}R_{t}}\;\mathrm{or}\;\bar{L_{b}R_{b}}\\ 0, & \mathrm{otherwise} \end{array}\right.$$

(9) $${\hat{P}}^{S}\left(x\right)=\left\{\begin{array}{cc} 1, & \mathrm{if}\;(x)\;\mathrm{on}\;\bar{LR}\\ 0, & \mathrm{otherwise} \end{array}\right.$$

where $\hat{P}\left(x\right)$ represents a random position in $\hat{P}$.

$M^{L}$ receives the vertebra image to estimate the PAFs $P^{L}$ and confidence maps $C_{i=1,2,3,4}^{L}$ for the landmarks. Likewise, $M^{S}$ predicts the confidence maps $C_{i=1,2}^{S}$ and PAFs $P^{S}$ for the landmarks of the sacrum. Then, through the loss functions $L_{M^{L}}$ and $L_{M^{S}}$, we trained $M^{L}$ and $M^{S}$, respectively.

(10) $$L_{M^{L}}=\frac{1}{4}\sum_{i=1}^{4}\left|\right|{\hat{C}}_{i}^{L}-C_{i}^{L}{\left|\right|}_{2}+\alpha ||{\hat{P}}^{L}-P^{L}{\left|\right|}_{2}$$

(11) $$L_{M^{S}}=\frac{1}{2}\sum_{i=1}^{2}\left|\right|{\hat{C}}_{i}^{S}-C_{i}^{S}{\left|\right|}_{2}+\beta ||{\hat{P}}^{S}-P^{S}{\left|\right|}_{2}$$

where we used α = 0.001 and β = 0.01, defined experimentally.

## 4. Experiments

This section defines the dataset used for our experiments and provides the experimental settings, along with the ablation studies on the proposed methods. Subsequently, the proposed network is compared to the previous work [5], which was modified to fit landmark detection.

### 4.1. Experimental Setup

We constructed a lateral view of the lumbar X-ray image dataset utilizing NHANES II’s lumbar X-ray dataset [26] from the National Library of Medicine and the BUU Spine Dataset [27] from Burapha University. Our dataset consisted of 1524 images, from which we used 976 images for training, 244 for validation, and 304 for testing, where we converted all images to grayscale. Moreover, we used the test set to yield all experimental results.

For training Pose-Net, we used the Adam optimizer [28] with a learning rate of $1\times 10^{-4}$ and a batch size of 16. Moreover, for stable learning, we used a learning rate scheduler, which reduced the learning rate linearly. We conducted training up to 200 epochs, but if the validation loss ceased to decrease for 30 epochs, we stopped it prematurely. For training M-Net, we used a batch size of 32. Similar to Pose-Net, we trained M-Net for up to 250 epochs and stopped early if the value of the validation loss did not drop for 25 epochs. All other settings were the same as for Pose-Net learning. We used the Pytorch v1.11.0 framework and CUDA v11.3, with a single NVIDIA RTX 3090 device, for conducting all experiments.

### 4.2. Experiments of Center Detector

In this section, we generated all the experimental results by mapping center coordinates created through Pose-Net to each input image $\in R^{512\times 512}$ of Pose-Net. First, we performed an experiment based on changing the kernel size of the convolution layer before applying random spine cutout augmentation. We increased the 7 × 7 convolution filter to 13 × 13 in the existing Pose-Net structure [5] to reduce the imprecise detection of all vertebrae centers by making the convolution filter scan more widely, including nearby vertebrae. Therefore, we performed a quantitative and qualitative comparison of the effect of changing the size of the convolution filter on detecting the centers of the lumbar vertebrae and sacrum. In all quantitative comparisons of center detection, an outlier refers to the proportion of images that do not include some landmarks in the image after pre-processing (i.e., cropping) using center coordinates generated by Pose-Net. The values in Table 2, Table 3 and Table 4 represent the average pixel distance errors (and standard deviation) between the predicted center coordinates and the ground truth of the center coordinates. In addition, (Inlier) in the first column of each table denotes the results except for outlier cases, while (All) denotes all results with outlier cases.

**Kernel size:**Table 2 shows the center distance error according to the changing of the kernel size. In Table 2, comparing the kernel sizes of Pose-Net, 7 × 7 and 13 × 13, the average distance error value of the latter is slightly higher than the former, but the outlier ratio of the latter is less by 1% than the former. In this work, we used Pose-Net to locate rough areas for cropping the target vertebra by detecting its center. Accordingly, to ensure a lower outlier ratio rather than a lower distance error on average, we adjusted the kernel size from 7 × 7 to 13 × 13.

**Figure 10:**
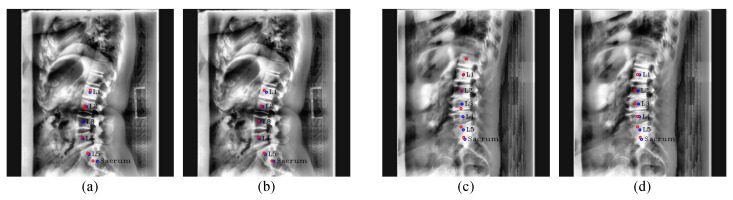
Inference results between kernel size of Pose-Net. (**b**,**d**) are Pose-Net results with the kernel size of 13 × 13, while (**a**,**c**) are Pose-Net results with the kernel size of 7 × 7. The red circles denote the predicated centers of L1, L2, L3, L4, L5, and the sacrum in order from the top, and blue circles and labels denote the ground truth of the centers.

Figure 10a shows the outlier result that occurred by incorrectly predicting the center of L2 in the channel as the center of L3. Furthermore, in Figure 10c, Pose-Net incorrectly predicted the middle of L3 and L4 as the center of L3 by recognizing L3 and L4 as one vertebra, which were deformed due to compression fractures. On the other hand, Figure 10b,d show that Pose-Net using the modified kernel size rather than 7 × 7 accurately predicted the centers of each vertebra. Here, the resolution of the Pose-Net output was 64 × 64 pixels, and the average distance of ground truth center coordinates between adjacent vertebrae was around 7 pixels. Therefore, when the kernel size is 7 × 7, the convolution filter barely sees the nearby vertebrae when scanning the center of each vertebra, and the rational center detection ability from surrounding information is insufficient. Consequently, we increased the kernel size of 7 × 7 to 13 × 13 in Pose-Net for the following experiments, which was around twice 7 × 7, enabling most of the neighboring vertebrae to be seen, allowing center detection performance better even if the shape of the vertebra was deformed.

**Random spine cutout (RSC):** Before we verify the effectiveness of RSC, we compared the performance of Pose-Net between the size of the RSC region because an increased similarity between the size of the RSC region and an actual occluded vertebra situation may have a more significant effect on RSC. When the lengths of the width and height of the RSC region were (1.0, 0.8, 0.6, 0.4)-times the width and height of each vertebra, respectively, we compared the performance of Pose-Net.

In Table 3, the distance error is typically low when the region of RSC is large. Cutting most of the vertebra area caused Pose-Net to learn strictly by increasing the loss value, and as a result, it made Pose-Net detect the center more precisely, while black background pixels might be considered as a masked vertebra and cause an outlier ratio increase. When the RSC region was too small (e.g., ratio of 0.4), both distance errors and outlier ratio increased. Therefore, we selected a ratio of 0.6, which had a relatively low distance error and the lowest outlier ratio.

We quantitatively and qualitatively compared the performance of the conventional cutout [8] and RSC. When using the conventional cutout, we set the region of the cutout similar to that of the RSC region.

Table 4 shows the benefit of RSC. In Table 4, we can see that training Pose-Net with RSC and the conventional cutout showed good performance in the distance error. However, unlike RSC, when using the conventional cutout only, the outlier ratio was higher than without the cutout. Since occlusion is a common feature of X-ray images, both RSC and the conventional cutout were effective in reducing the distance errors. However, our RSC on the lumbar vertebrae (L1–L4) was more effective in drastically decreasing the distance error and outlier ratio.

**Figure 11:**
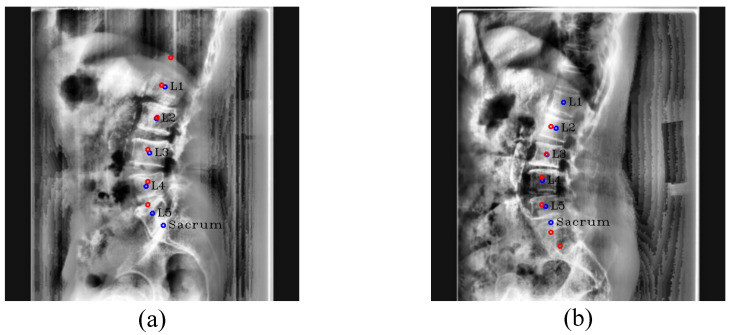
Center detection failure cases. (**a**,**b**) are outlier cases caused by the similar appearance of L5 and the sacrum. The red circles denote the predicated centers of L1, L2, L3, L4, L5, and the sacrum in order from the top, and blue circles and labels denote the ground truth of the centers.

However, about 2% of outliers still existed even with RSC. These are difficult cases for both Pose-Net and radiologists to localize the vertebrae precisely, as shown in Figure 11. Our Pose-Net predicted L5 as the sacrum (S1) in Figure 11a, yielding all predictions incorrect. In this case, because L5 can be seen as S1 due to lumbosacral transitional vertebrae [29], radiologists also cannot easily differentiate one from the other. Similarly, in Figure 11b, our Pose-Net predicted S1 as L5, and it had difficulty distinguishing S1 from L5 based on the X-ray image.

**Figure 12:**
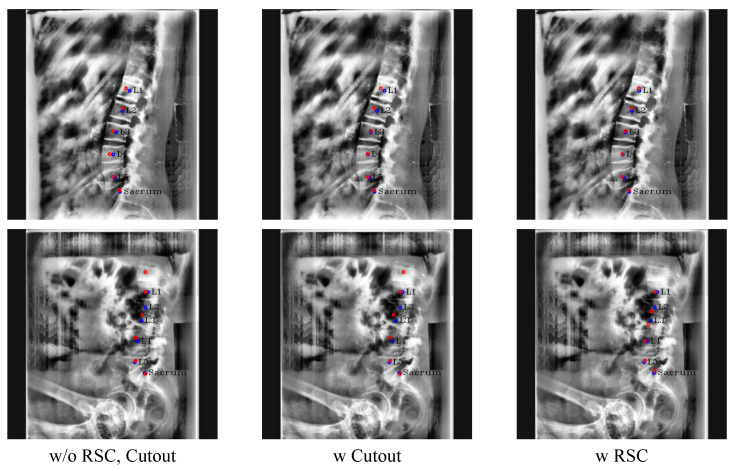
Results of ablation study with the conventional cutout and RSC. The red circles denote the predicated centers of L1, L2, L3, L4, L5, and the sacrum in order from the top, and blue circles and labels denote the ground truth of the centers.

We also compared RSC and the conventional cutout qualitatively in Figure 12. As illustrated in the first row of Figure 12, the centers of L3 and L4 were more precisely localized when either RSC or the conventional cutout was employed compared to the results without cutout augmentation. Where the center area was occluded like L1, the predicted center of L1 was skewed to the left when using the conventional cutout technique. In contrast, in the case of RSC, the center of L1 was accurately predicted. Moreover, only RSC can drive Pose-Net to accurately detect sequential L1 to L3, even when it is difficult to visually distinguish the boundary between L2 and L3, as shown in the second row of Figure 12. Therefore, it is evident that RSC is superior to the conventional cutout technique in the lumbar X-ray image and can make Pose-Net more robust in circumstances where the vertebrae are not clearly visible by cutting only the lumbar vertebrae.

### 4.3. Experiments of Landmark Detector

In this section, we validated whether learning the local and morphological information by CoordConv [10] and PAFs was efficient in M-Net to predict landmarks accurately. We obtained all results of landmark detection by mapping M-Net’s inference results to the original input image. And in all quantitatively comparing landmark detection, we excluded the outlier results of Pose-Net and used a relative distance error (RD) to evaluate the predicted accuracy based on vertebra size besides the pixel distance error (D). The relative distance error of lumbar vertebrae is formulated by Equations (12)–(14), where gt is the ground truth of the target landmark, pred is the result of detecting the corresponding landmark, $pred_{x}$ is the x-coordinate of pred, and *h* and *v* are the lengths of horizontal and vertical lines that connect gt and nearby landmarks with gt, respectively. For the sacrum, it is formulated using Equation (15), where *l* is the length of the sacrum.

(12) $$r_{x}=\frac{pred_{x}-gt_{x}}{h}$$

(13) $$r_{y}=\frac{pred_{y}-gt_{y}}{v}$$

(14) $$\mathrm{relative}\;\mathrm{distance}\;\mathrm{error}\left(\%\right)=100\times \sqrt{{r_{x}}^{2}+{r_{y}}^{2}}$$

(15) $$\mathrm{relative}\;\mathrm{distance}\;\mathrm{error}\left(\%\right)=100\times \frac{||pred-{gt||}_{2}}{l}$$

**CoordConv:** We utilized CoordConv in M-Net’s encoding layers and analyzed its effectiveness along with the optimal number of CoordConvs.

Table 5 shows that utilizing one CoordConv in the encoding layer of M-Net (CC(1)) significantly increased the detection accuracy of the sacrum landmarks while decreasing the detection accuracy of lumbar vertebrae rather than without CoordConv. When utilizing CoordConv in two encoding layers (CC(2)), the detection accuracy of the both lumbar vertebrae and the sacrum was higher than without CoordConv. It is evident that CC(1) is only practical for the sacrum with a few landmarks, but CC(2) is beneficial to both the sacrum and the lumbar vertebrae. Consequently, in subsequent experiments, we employed CC(2).

**Figure 13:**
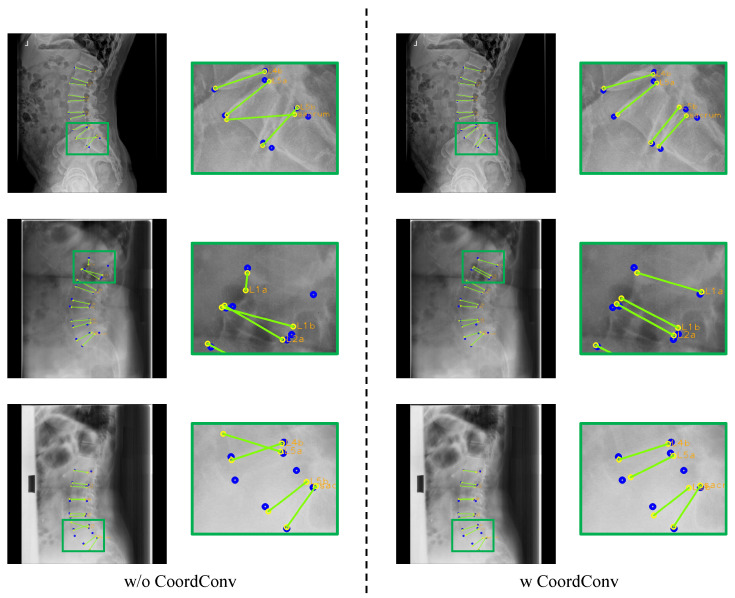
Results of landmark detection depending on CoordConv. The blue circles represent the ground truth, while yellow circles with labels represent predicted results and the green lines represent the line connecting the predicted landmarks of an endplate.

Figure 13 shows the effect of CoordConv. The boundary between L5 and the sacrum is hard to identify in the first row of Figure 13, and when without utilizing CoordConv, M-Net incorrectly predicted the top-left landmark of L5 as the left landmark of the sacrum. Furthermore, when the boundaries of L1 and L5 are invisible, as in Rows 2 and 3 of Figure 13, respectively, the case without CoordConv predicted incorrect locations as landmarks because it predicts based simply on visual information. When employing CoordConv, however, M-Net can use not only visual data, but also location data to predict the landmark. Consequently, utilizing CoordConv allows the network to learn the location distribution and derive accurate landmark detection even when landmarks are invisible.

**Part affinity fields (PAFs):** We let M-Net predict PAFs to learn appearance information about the vertebrae and compared quantitatively and qualitatively whether this benefits detecting landmarks. Furthermore, we conducted an ablation study to determine which width is suitable for the ground truth.

**Figure 14:**
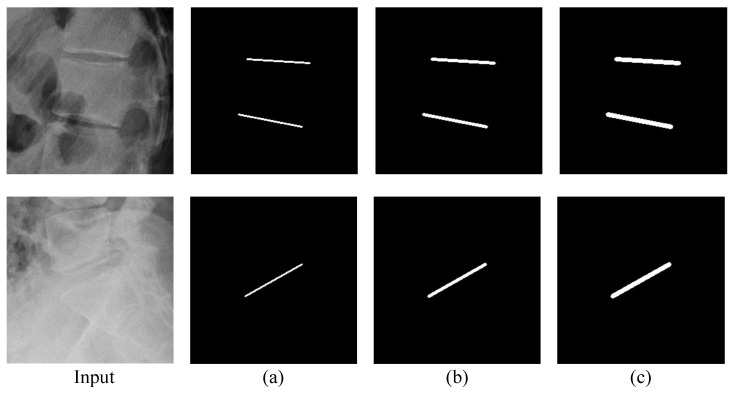
Example of the ground truth of PAFs by width: (**a**) 2 pixels, (**b**) 4 pixels, and (**c**) 6 pixels.

According to the ground truth width of PAFs, as depicted in Figure 14, we measured the performance of M-Net (Table 6). When the width of PAFs was thin such as 2 pixels, there was no significant performance improvement. This is because M-Net cannot adequately learn the morphological information of the vertebrae since the loss value of PAFs is low. However, when the width was sufficiently thick, such as 6 pixels, the PAFs loss value became large and M-Net learned with the aim of PAF prediction rather than landmark detection. This caused low performance. Therefore, we selected PAFs with a width of 4 pixels as the ground truth, which improved the overall performance significantly. Comparing the performance with and without PAFs demonstrated that predicting PAFs improved the accuracy of landmark detection.

**Figure 15:**
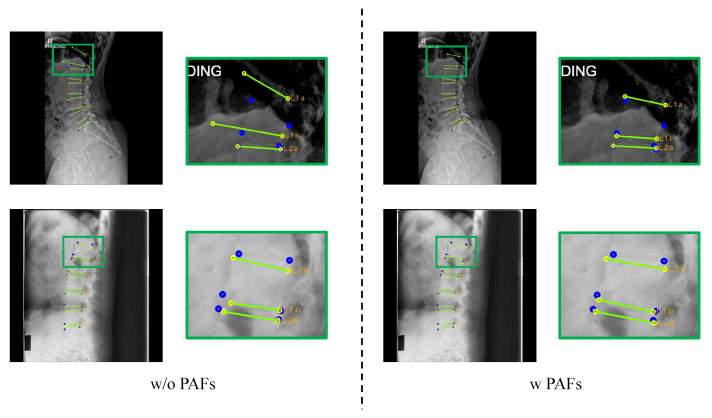
Results of landmark detection with or without PAFs. The blue circles represent the ground truth, while yellow circles with labels represent predicted results, and the green lines represent the line connecting the predicted landmarks of an endplate.

Figure 15 shows the effect of predicting PAFs. When without PAFs, M-Net makes an anomalous prediction, as shown in the first row of Figure 15, when L1 is blackened. Moreover, in the second row of Figure 15, the lengths of L1a and L1b are wildly different, and the two lines are not parallel. Prediction in this way might cause misdiagnosis when making a diagnosis about spine alignment. However, when utilizing PAFs, M-Net predicts more accurately based on the learned vertebral shape (i.e., L1a and L1b have similar lengths and are parallel). Without PAFs, it is hard to detect landmarks precisely when the corner points of the vertebra are not distinct, relying only on the visuals of the input image. When M-Net predicts PAFs, it is possible to have accurate detection because the morphological features to predict PAFs are included in the process of landmark detection.

**CoordConv and PAFs:** We both quantitatively and qualitatively analyzed if M-Net takes full advantage of both CoordConv and PAFs. Table 7 shows the landmark detection errors according to using them.

In Table 7, we observe that the accuracy was significantly high for the vertebrae located in the upper region of the lumbar vertebrae (L1 and L3) when using PAFs and for the lower half of the lumbar vertebrae (L4 and L5) when using CoordConv. PAFs based on morphological information outperformed CoordConv for the upper section of the lumbar vertebrae, where its landmark locations differ substantially from the body to body. In contrast, CoordConv outperformed PAFs for the lower lumbar area, where the landmarks are located at similar positions for all bodies. Employing both CoordConv and PAFs showed improved performances compared to using CoordConv alone for the upper section of the lumbar vertebrae and using PAFs alone for the lower section. Furthermore, it showed the lowest total average distance error. Consequently, M-Net detected the landmark more accurately by utilizing the benefits of both CoordConv and PAFs.

**Figure 16:**
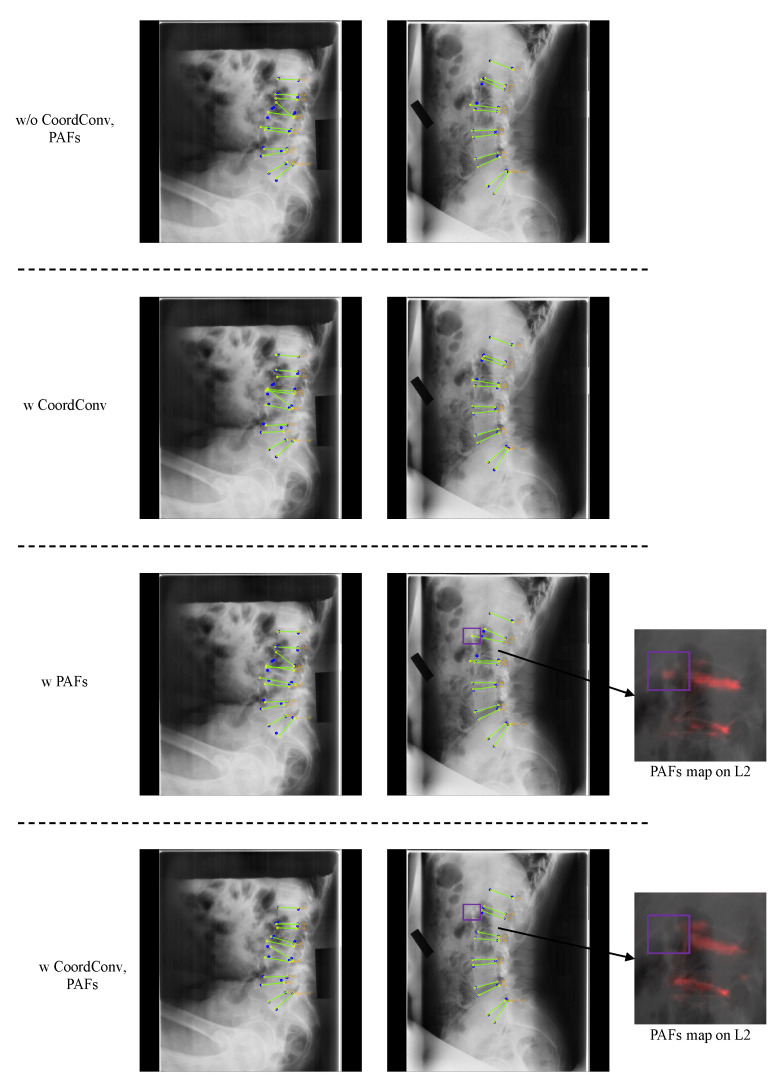
Results of ablation study with CoordConv and PAFs. The blue circles represent the ground truth, while yellow circles with labels represent predicted results, and the green lines represent the line connecting the predicted landmarks of an endplate.

The effect of employing both CoordConv and PAFs can be seen more intuitively in Figure 16. In the first column, the spine area is very faint and the left and right sides of L3 are asymmetric with an abnormal shape. In this case, there was no significant performance improvement when using either CoordConv or PAFs. When using only CoordConv, L3b was detected more accurately, but L2a, L2b, and L3a were still incorrectly detected. Due to the abnormal shape of L3, the locations of landmarks were unusual, so using position information alone was ineffective for landmark detection. When using PAFs alone, L2a prediction worsened, and L2b, L3a, and L3b were still not properly detected. Again, due to the abnormal shape of L3, it is challenging to predict PAFs, and inaccurately predicted PAFs negatively affect predicting L3 landmarks. In the second column, the L2a was predicted close to the ground truth when using only CoordConv rather than without CoordConv and PAFs, but the prediction of L2b was not improved. PAFs of L2 were incorrectly predicted when only PAFs were employed, resulting in worse detection results than those without PAFs, as shown in the purple box of the third column. However, when utilizing both CoordConv and PAFs, L2b was detected more accurately, recognizing the morphological correlation between L2a and L2b based on the position information. Consequently, when employing both CoordConv and PAFs, all landmarks were predicted well since PAFs can be predicted more precisely with CoordConv. Therefore, it is possible to overcome the problem of incorrectly predicted PAFs by using CoordConv, and landmarks were precisely detected by combining the benefits of both methods.

We also compared the inference times of the methods. Table 8 shows the average time for detecting all landmarks from each test set image. When using only PAFs, the inference time only increased by 0.2% because it only needed to output an additional one-channel output predicting PAFs with M-Net. Furthermore, when employing PAFs, the inference time raised slightly while the landmark detection accuracy was greatly enhanced, as seen in Table 7. Employing CoordConv was also effective for detecting landmarks with an average inference time of 21.87 ms, which is not an issue in real life.

Finally, we show how well our network performed by a box plot and an outlier ratio table using relative distance errors. We define an outlier case when the average relative distance error of all landmarks in each vertebra is higher than 20%, which is farther than the typical distance between neighboring landmarks of vertebrae (e.g., the distance between the left bottom landmark of L1 and the left top landmark of L2). Table 9 shows the ratio of outlier cases.

Table 9 shows the average outlier ratio of each vertebra. In Table 9, the outlier ratios are low for L2–L5, but very high for L1 and the sacrum. L1 is frequently occluded by thoracic structures or deformed due to a severe compression fracture, and this results in a higher outlier ratio than other lumbar vertebrae. L5 and S1 are too confused to differentiate one from the other when the lumbosacral transitional vertebrae exist. Accordingly, the outlier ratio of the sacrum landmarks is exceptionally high and even radiologists cannot easily find the exact position of the sacrum.

**Figure 17:**
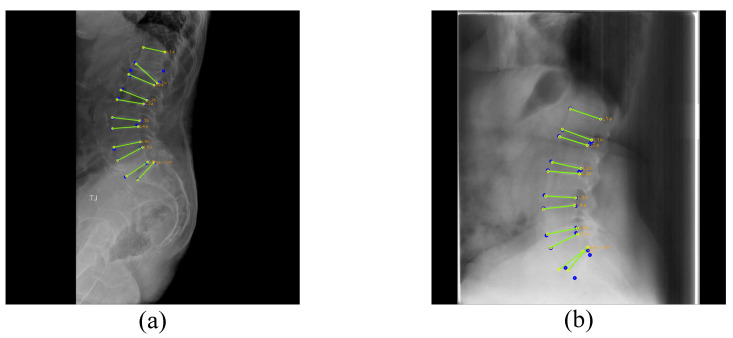
Landmark detection failure cases. (**a**,**b**) are outlier cases caused by severe compression fracture and occlusion, respectively. The blue circles represent the ground truth, while yellow circles with labels represent predicted results, and the green lines represent the line connecting the predicted landmarks of an endplate.

**Figure 18:**
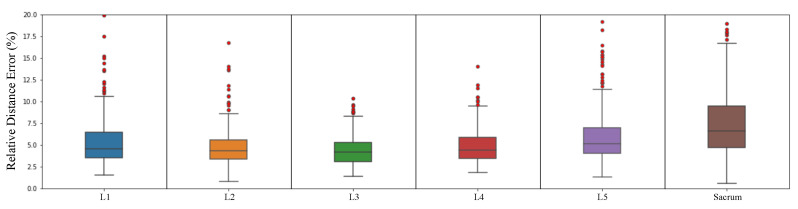
Box plot of landmark relative distance error except for outliers.

These several outlier cases are shown in Figure 17. Our M-Net detected some T12 landmarks as L1 landmarks in Figure 17a because the L1 compression fracture is so severe that the vertebra does not form a normal shape. In Figure 17b, M-Net incorrectly predicted the sacrum because L5 and the sacrum areas are not apparent. For many challenging cases, our network had an average landmark detection accuracy of 98.38%. The relative distance error box plot for each vertebra except outliers is presented in Figure 18. This shows that our network had a low average relative distance error across every vertebra.

### 4.4. Comparison to the Previous Work

We compared the performances of our method and Kim et al. [5] as the previous method quantitatively and qualitatively. For a fair comparison, the network of Kim et al. was modified from a segmentation task to a landmark detection task, and we considered the results of lumbar vertebrae since there was no result for the sacrum in Kim et al.

**Figure 19:**
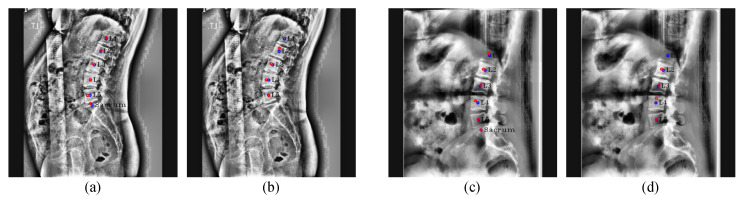
Results of center detection. Our results are (**a**,**c**), and the results of Kim et al. [5] are (**b**,**d**). The red circles denote the predicated centers of L1, L2, L3, L4, L5, and the sacrum in order from the top, and the blue circles and labels denote the ground truth of the center of each vertebra.

**Center detector:**Table 10 shows that our overall center detection ability was superior to Kim et al., particularly in terms of the outlier ratio and the distance error of L1. When localizing the center of each lumbar vertebra, Kim et al. generated a center confidence map of 1 channel and extracted the center coordinates of the lumbar vertebrae. At this time, in the case of L1, the confidence score was low when obscured by thoracic structures, as shown in the Discussion Section of [5], making it challenging to extract local maximum coordinates through the post-processing of Kim et al. Moreover, Kim et al. eliminated the confidence map when the confidence score was too low, and there were many outlier cases in which the center coordinates were not extracted for L1 because of the elimination, as shown in Figure 19. In addition, the predicted location of the centers was slightly different from the ground truth. However, our method can detect more accurately using a wide convolution filter size and RSC. Furthermore, even when some of the target center coordinates were not extracted, we used the multi-channel confidence map including the confidence map with low confidence scores during the post-processing step to ensure that all center coordinates were extracted.

**Figure 20:**
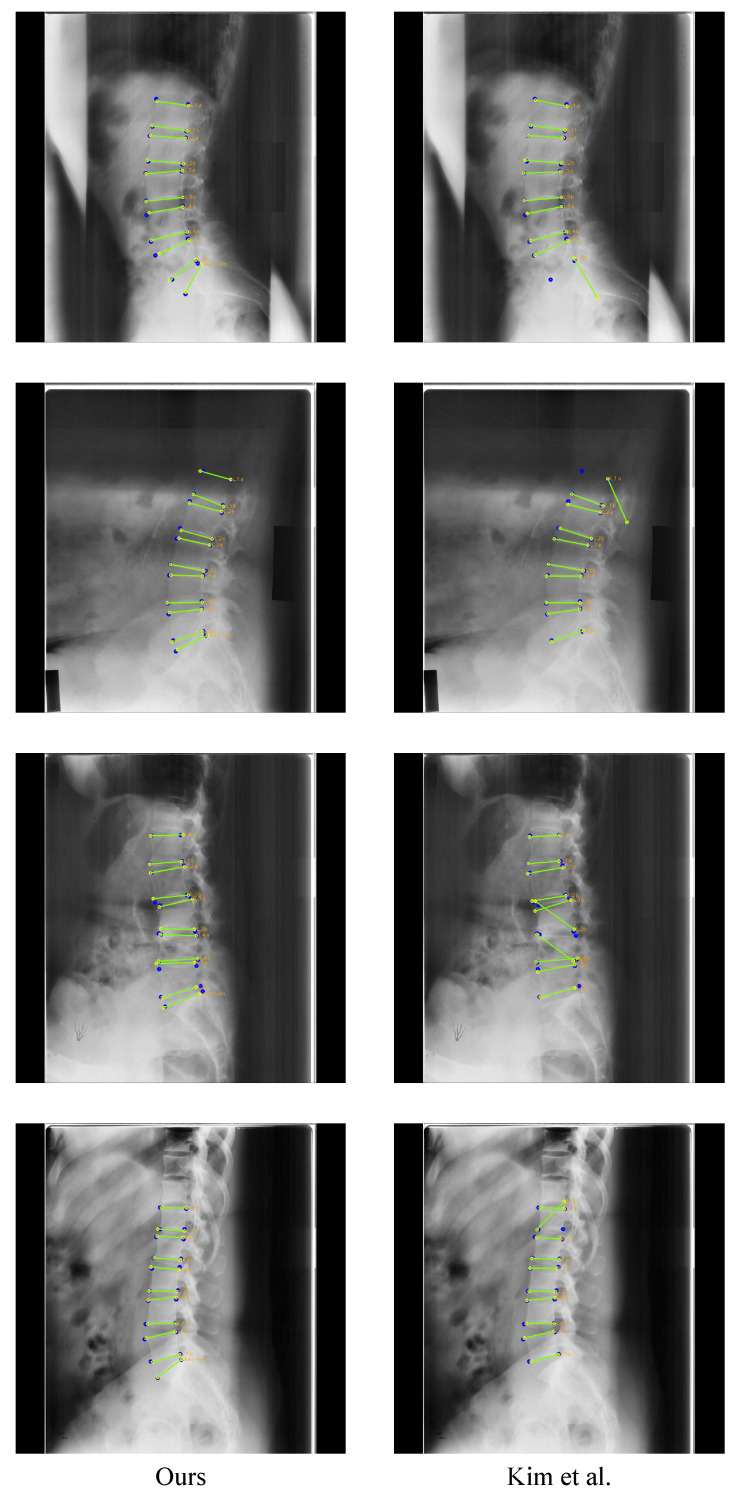
Example of landmark detection result of ours and Kim et al. [5]. The blue circles represent the ground truth, while yellow circles with labels represent predicted results, and the green lines represent the line connecting the predicted landmarks of an endplate.

**Landmark detector:** As shown in Table 11, our method outperformed Kim et al. in every vertebral landmark detection, except L3. Especially, our network showed lower distance errors of 16.3259 pixels and 10.3488 pixels, respectively, for L1 and L2 compared to Kim et al. When landmarks were barely visible, such as L5 in the first row and L1–L2 in the second row of Figure 20, unlike Kim et al., which detected landmarks solely based on visuals, our method was much better as it predicted landmarks using location and morphological information through CoordConv and PAFs. Furthermore, Kim et al. often incorrectly predicted vertebral landmarks, even when the landmarks were a little occluded, as in the third row and fourth row. As a result, the standard deviation of the distance error values was very high, as shown in Table 11, indicating that it was not detected reliably. However, thanks to CoordConv and PAFs, our method achieved better landmark detection accuracy and reliability.

**Figure 21:**
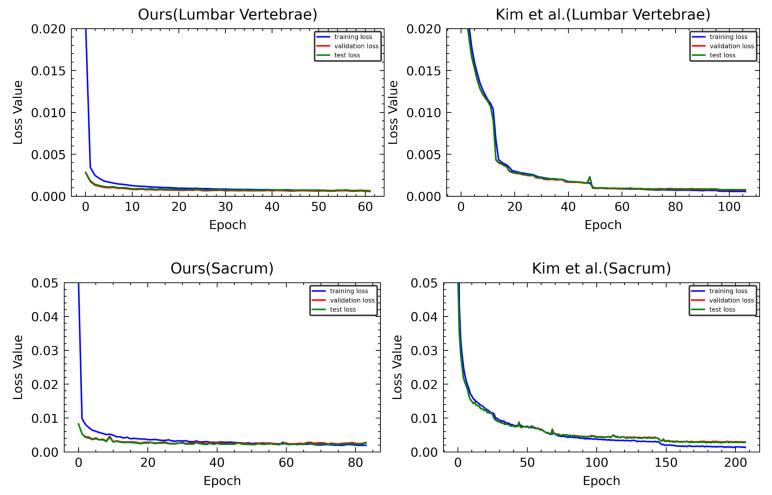
Loss graphs of ours and Kim et al. [5]. The first row is a loss graph of M-Net predicting the landmark of the lumbar vertebrae, and the second row is a loss graph of M-Net predicting the landmark of the sacrum.

Furthermore, as in Figure 21, our method had a very fast convergence of loss and completed learning in a few epochs by early stopping, whereas Kim et al. had a low convergence speed and needed more epochs for learning.

## 5. Conclusions

In this paper, we presented a novel two-stage network for detecting the landmarks of the lumbar vertebrae and sacrum on X-ray images. The proposed network detected landmarks from the vertebral images, which were cropped from the zero-padded input image using the detected center of each vertebra. In the center detection process, we expanded the receptive fields of the network to perform more accurate detection. Moreover, our proposed random spine cutout augmentation technique made the network perform detection more robustly on X-ray images, reflecting the properties of X-rays, which are often partially obscured. Additionally, we used CoordConv and part affinity fields to improve the accuracy of landmark detection by learning the distribution of landmark positions and the structural features of the vertebrae.

Our experiments showed that using random spine cutout, which directly cuts a random vertebra, was more effective in increasing the center detection accuracy of each vertebra than using the conventional cutout in a lumbar X-ray image. Furthermore, it also demonstrated that learning location information through CoordConv helped to detect occluded landmarks, and the prediction of PAFs was effective in enhancing the detection performance by recognizing the shape features of the vertebra. Finally, the landmark detection accuracy of our proposed network was 98.38%.

However, there were some failure cases because of severe compression fractures. This can be overcome by using the image transform augmentation technique, which makes a normal vertebra look like a compression fracture, and this remains as future work.

The proposed network can be utilized to diagnose spondylolisthesis at the L5–S1 level since it detects landmarks on the upper end plate of the S1, in addition to quantifying spinal alignment, scoliosis, the compression factor, and degenerative change. 

## Figures and Tables

**Table 1:** Average pixel values inside each vertebra (and the standard deviation).

Vertebra	Pixel Value
L1	137.11(30.99)
L2	132.20(29.65)
L3	130.83(28.02)
L4	140.09(25.58)
L5	163.02(25.62)

**Table 2:** Comparison of center distance error of Pose-Net according to kernel size change. The value having the lowest distance error for each column in (Inlier) is marked in bold and underlined for (All).

Kernel Size	L1	L2	L3	L4	L5	Sacrum	Total	Outlier (%)
7 (Inlier)	6.2048(3.7175)	**5.6754(2.5657)**	**5.8699(2.4392)**	**5.9250(2.6028)**	6.5041(2.9050)	6.5863(3.8569)	**6.1276(3.0809)**	3.6184
7 (All)	7.3140(7.6024)	6.9445(7.9221)	7.1655(8.1824)	6.9841(7.5247)	7.4520(7.3884)	7.5333(7.5562)	7.2322(7.6934)	
7->13 (Inlier)	**6.1645(2.6868)**	6.7569(2.6437)	6.7818(2.5824)	6.6531(2.5095)	**6.3572(2.7250)**	**6.3670(3.6241)**	6.5134(2.8260)	**2.6316**
7->13 (All)	7.2846(7.3810)	7.9211(7.6721)	7.9006(7.6783)	7.7393(7.5274)	7.4110(7.5285)	7.2813(7.3648)	7.5896(7.5210)	

**Table 3:** Comparison of center distance error of Pose-Net according to the size of the random spine cutout (RSC) region. The value having the lowest distance error for each column in (Inlier) is marked in bold and underlined for (All).

Ratio	L1	L2	L3	L4	L5	Sacrum	Total	Outlier (%)
1.0 (Inlier)	5.9408(2.6590)	**5.8152(2.3992)**	5.6638(2.4129)	6.0802(2.5584)	**5.7691(2.5759)**	6.5375(3.4117)	**5.9677(2.7031)**	2.9605
1.0 (All)	7.0061(7.3041)	6.9253(7.6269)	6.8778(8.1331)	7.2323(7.9045)	6.8937(7.9268)	7.6288(7.9528)	7.0940(7.8065)	
0.8 (Inlier)	5.9864(2.6269)	6.2773(2.4904)	**5.6182(2.3728)**	6.0225(2.4082)	6.0451(2.5293)	**6.1910(3.5926)**	6.0234(2.7071)	2.3026
0.8 (All)	6.8910(6.9462)	7.2230(7.0324)	6.6239(7.5476)	6.9955(7.3794)	7.0123(7.2798)	7.1463(7.7933)	6.9820(7.3280)	
0.6 (Inlier)	**5.9070(2.7222)**	6.1465(2.4507)	5.6808(2.3847)	**5.9585(2.5786)**	6.1200(2.6225)	6.8137(3.5138)	6.1044(2.7567)	**1.9737**
0.6 (All)	6.7998(6.9070)	7.0464(6.8417)	6.6200(7.1450)	6.9024(7.2187)	7.0595(7.1900)	7.7733(7.8280)	7.0336(7.1948)	
0.4 (Inlier)	6.1318(2.8215)	6.3584(2.5233)	5.8741(2.4999)	5.9819(2.6023)	6.0978(2.6394)	6.2191(3.6614)	6.1105(2.8205)	3.6184
0.4 (All)	7.4260(8.0499)	7.6042(7.6288)	6.9495(7.4526)	6.9735(7.2562)	7.0888(7.3214)	7.2202(7.8299)	7.2104(7.5883)	

**Table 4:** Ablation study of the conventional cutout (CO) and random spine cutout (RSC). The value having the lowest distance error for each column in (Inlier) is marked in bold and underlined for (All).

	L1	L2	L3	L4	L5	Sacrum	Total	Outlier (%)
w/o (RSC, CO) (Inlier)	6.1645(2.6868)	6.7569(2.6437)	6.7818(2.5824)	6.6531(2.5095)	6.3572(2.7250)	6.3670(3.6241)	6.5134(2.8260)	2.6316
w/o (RSC, CO) (All)	7.2846(7.3810)	7.9211(7.6721)	7.9006(7.6783)	7.7393(7.5274)	7.4110(7.5285)	7.2813(7.3648)	7.5896(7.5210)	
w CO (Inlier)	6.3759(3.0089)	6.3484(2.9113)	6.1966(2.5607)	**5.8308(2.5506)**	6.2939(2.7560)	**6.2061(4.4343)**	6.2086(3.1060)	2.9605
w CO (All)	7.5616(7.6095)	7.5371(7.8315)	7.3814(7.9178)	7.0085(8.1010)	7.4556(8.1073)	7.3054(8.6486)	7.3749(8.0335)	
w RSC (Inlier)	**5.9070(2.7222)**	**6.1465(2.4507)**	**5.6808(2.3847)**	5.9585(2.5786)	**6.1200(2.6225)**	6.8137(3.5138)	**6.1044(2.7567)**	**1.9737**
w RSC (All)	6.7998(6.9070)	7.0464(6.8417)	6.6200(7.1450)	6.9024(7.2187)	7.0595(7.1900)	7.7733(7.8280)	7.0336(7.1948)	

**Table 5:** Ablation study of CoordConv(CC). CC(1) means using CoordConv in the first encoding layer of M-Net, and CC(2) means using CoordConv in the first two encoding layers, as shown in Figure 8. The lowest value for each column in distance error (D) is marked in bold and underlined in relative distance error (RD).

	L1	L2	L3	L4	L5	Sacrum	Total
w/o CC (D)	13.4629(14.5039)	10.8182(6.1662)	10.2746(5.6941)	10.6966(4.8587)	13.2534(8.1749)	22.7648(20.0812)	13.5451(12.1363)
w/o CC (RD)	6.4143(7.0429)	4.9426(2.9314)	4.6324(2.6764)	4.9216(2.4185)	6.3256(4.2349)	9.5112(8.7448)	6.1246(5.5049)
w CC(1) (D)	13.8476(15.5072)	10.7795(4.8753)	10.5213(5.4359)	10.9129(4.7082)	13.5546(7.9351)	**21.3077(17.3570)**	13.4873(11.2722)
w CC(1) (RD)	6.5605(7.5749)	4.9090(2.3354)	4.7562(2.9093)	4.9928(2.2583)	6.4696(4.0365)	8.8381(7.7252)	6.0877(5.2320)
w CC(2) (D)	**13.2129(13.9569)**	**10.4010(5.0925)**	**10.1377(4.6663)**	**10.6879(5.3652)**	**12.8991(6.6441)**	22.1391(16.5678)	**13.2463(10.7381)**
w CC(2) (RD)	6.3072(7.0595)	4.7426(2.4540)	4.5529(2.1768)	4.9061(2.4465)	6.1400(3.3254)	9.1456(7.3295)	5.9657(4.9308)

**Table 6:** Performance according to the width of the ground truth of PAFs. The lowest value for each column in distance error (D) is marked in bold and underlined in relative distance error (RD).

	L1	L2	L3	L4	L5	Sacrum	Total
w/o PAFs (D)	13.4629(14.5039)	10.8182(6.1662)	10.2746(5.6941)	**10.6966(4.8587)**	**13.2534(8.1749)**	22.7648(20.0812)	13.5451(12.1363)
w/o PAFs (RD)	6.4143(7.0429)	4.9426(2.9314)	4.6324(2.6764)	4.9216(2.4185)	6.3256(4.2349)	9.5112(8.7448)	6.1246(5.5049)
w PAFs-width 2 (D)	13.9451(14.8208)	10.6166(5.6704)	9.9683(3.9839)	10.9204(5.0152)	13.4877(8.9793)	22.3652(18.6160)	13.5505(11.7209)
w PAFs-width 2 (RD)	6.6628(7.4286)	4.8374(2.6447)	4.4580(1.9171)	5.0171(2.5283)	6.4009(4.4703)	9.2942(8.2189)	6.1117(5.4070)
w PAFs-width 4 (D)	**12.2740(9.0398)**	**10.4992(5.8889)**	**9.8556(5.0278)**	10.7250(4.8108)	13.2953(7.5819)	**21.6285(17.7035)**	**13.0463(10.2522)**
w PAFs-width 4 (RD)	5.8339(4.6423)	4.8065(3.0228)	4.4455(2.7755)	4.9670(2.4390)	6.3455(3.8456)	8.9756(7.7470)	5.8957(4.7022)
w PAFs-width 6 (D)	13.7257(17.3613)	10.6346(6.0329)	9.9886(3.9369)	10.9204(5.9805)	13.3778(9.0107)	21.7235(17.0877)	13.3951(11.9373)
w PAFs-width 6 (RD)	6.5436(8.4218)	4.8677(3.0381)	4.4686(1.8798)	5.0277(2.8025)	6.3426(4.4093)	9.0349(7.7065)	6.0475(5.5391)

**Table 7:** Ablation study of CoordConv(CC) and PAFs. The lowest value for each column in distance error (D) is marked in bold and underlined in relative distance error (RD).

	L1	L2	L3	L4	L5	Sacrum	Total
w/o (CC, PAFs) (D)	13.4629(14.5039)	10.8182(6.1662)	10.2746(5.6941)	10.6966(4.8587)	13.2534(8.1749)	22.7648(20.0812)	13.5451(12.1363)
w/o (CC, PAFs) (RD)	6.4143(7.0429)	4.9426(2.9314)	4.6324(2.6764)	4.9216(2.4185)	6.3256(4.2349)	9.5112(8.7448)	6.1246(5.5049)
w CC (D)	13.2129(13.9569)	10.4010(5.0925)	10.1377(4.6663)	**10.6879(5.3652)**	**12.8991(6.6441)**	22.1391(16.5678)	13.2463(10.7381)
w CC (RD)	6.3072(7.0595)	4.7426(2.4540)	4.5529(2.1768)	4.9061(2.4465)	6.1400(3.3254)	9.1456(7.3295)	5.9657(4.9308)
w PAFs (D)	**12.2740(9.0398)**	10.4992(5.8889)	**9.8556(5.0278)**	10.7250(4.8108)	13.2953(7.5819)	21.6285(17.7035)	13.0463(10.2522)
w PAFs (RD)	5.8339(4.6423)	4.8065(3.0228)	4.4455(2.7755)	4.9670(2.4390)	6.3455(3.8456)	8.9756(7.7470)	5.8957(4.7022)
w (CC, PAFs) (D)	12.9856(12.2581)	**10.2726(4.3833)**	9.9563(4.1797)	10.6939(4.4190)	12.9161(6.7633)	**21.3219(16.1635)**	**13.0244(10.0280)**
w (CC, PAFs) (RD)	6.1385(5.8977)	4.7145(2.1368)	4.4771(2.2258)	4.9122(2.1481)	6.1483(3.4313)	8.8486(7.2449)	5.8732(4.5841)

**Table 8:** Average landmark detection time according to the usage of CoordConv and PAFs. Increase Rate denotes the relative increment in inference time compared to w/o (CoordConv, PAFs).

	w/o (CoordConv, PAFs)	w CoordConv	w PAFs	w (CoordConv, PAFs)
Average Inference Time (ms)	12.98(0.24)	21.71(0.37)	13.01(0.27)	21.87(0.53)
Increase Rate (%)	0	67.3	0.2	68.5

**Table 9:** Average outlier ratio for each vertebra.

	L1	L2	L3	L4	L5	Sacrum
Outlier (%)	2.68	0	0.34	0.34	0.67	5.70

**Table 10:** Comparison of center detection performance between ours and Kim et al. [5]. The value having the lowest distance error for each column in (Inlier) is marked in bold and underlined for (All).

	L1	L2	L3	L4	L5	Total	Outlier (%)
Ours (Inlier)	**5.9070(2.7222)**	6.1465(2.4507)	**5.6808(2.3847)**	**5.9585(2.5786)**	6.1200(2.6225)	**5.9626(2.5567)**	**1.9737**
Ours (All)	6.7998(6.9070)	7.0464(6.8417)	6.6200(7.1450)	6.9024(7.2187)	7.0595(7.1900)	6.8856(7.0548)	
Kim et al. [5] (Inlier)	11.1426(31.2408)	**5.8518(2.8318)**	6.2710(2.6000)	6.2763(2.6771)	**5.7273(2.5778)**	7.0538(14.3036)	4.6053
Kim et al. [5] (All)	18.8371(50.7964)	6.4124(5.5082)	6.7307(5.2587)	6.7293(5.1520)	6.0839(5.1002)	8.9587(23.6900)	

**Table 11:** Comparison of landmark detection performance between our method and Kim et al. [5]. The lowest value for each column in distance error (D) is marked in bold and underlined in relative distance error (RD).

	L1	L2	L3	L4	L5	Total
Ours (D)	**12.9856(12.2581)**	**10.2726(4.3833)**	9.9563(4.1797)	**10.6939(4.4190)**	**12.9161(6.7633)**	**11.3649(7.2139)**
Ours (RD)	6.1385(5.8977)	4.7145(2.1368)	4.4771(2.2258)	4.9122(2.1481)	6.1483(3.4313)	5.2781(3.5530)
Kim et al. [5] (D)	29.3115(105.0534)	20.6214(84.7178)	**9.6515(6.8754)**	11.1020(11.0167)	16.4756(21.1937)	17.4324(61.6963)
Kim et al. [5] (RD)	14.6237(54.6018)	9.8399(42.7799)	4.2791(2.7548)	4.9899(4.3484)	7.9765(10.7887)	8.3418(31.6554)

## Data Availability

Not applicable.
